# Recent advances in predicting, preventing, and managing postoperative delirium

**DOI:** 10.12703/r/12-19

**Published:** 2023-07-28

**Authors:** Owais Qureshi, Mary E Arthur

**Affiliations:** 1 Medical College of Georgia at Augusta University, Augusta, Georgia

**Keywords:** Postoperative delirium, prehabilitation, frailty syndrome, anesthesia

## Abstract

Postoperative delirium (POD) is a major public health problem associated with poor patient outcomes such as increased hospital lengths of stay, loss of functional independence, and higher mortality. Depending on the study, the reported incidence ranges from 5% to 65%, with the highest incidence in hip and cardiac surgery. Anesthesiologists should be familiar with the predisposing and precipitating factors of POD, particularly screening for preoperative cognitive impairment and frailty syndrome. Screening tools, for example, the Mini-Mental State Exam, Mini-Cog, 4 A's test for delirium screening, and Montreal Cognitive Assessment, can be used to assess for cognitive impairment and the Clinical Frailty Scale to assess for frailty syndrome. The Hospital Elder Life Program is the standard prevention protocol that is tried and tested in reducing the incidence of POD. Prehabilitation, lung protective strategies, pharmacologic agents such as ramelteon, a melatonin receptor agonist, glucocorticoids, dexmedetomidine, and non-pharmacologic agents, such as noise reduction strategies and the encouragement of nocturnal sleep, have all led to a decrease in the incidence of POD and are being studied for their efficacy. However, the data are inconclusive to date. Intraoperatively, preventing hypotension and blood pressure swings, ensuring adequate pain control and anesthetic depth, and using age-adjusted minimum alveolar concentration (MAC) titration reduce the incidence of POD. The incidence of POD using regional or general anesthesia is similar. In this narrative review, we will discuss the current understanding of the predictors, pathophysiology, prevention, and management of POD and identify areas of further research.

## Introduction

Postoperative delirium (POD) is a major public health problem associated with significantly worse patient outcomes, such as increased hospital lengths of stay, loss of functional independence, long-term post-traumatic stress disorder, and higher mortality 6–12 months after surgery^1^. The Diagnostic and Statistical Manual for Mental Disorders, Fifth Edition, defines POD as an acute confusional state with alterations in attention, awareness, and consciousness. It is reversible and has a fluctuating course characterized by two activity phenotypes, hypoactive and hyperactive^2^. The classic and most noticeable phenotype is the hyperactive type, where the patient is confused, hallucinating, and pulling on intravenous lines after surgery. However, hypoactive delirium is more common, which makes diagnosis quite tricky. The duration typically lasts hours to a few days. Less commonly, it may last weeks to months^3^. In this review, we will discuss the current understanding of the predictors, pathophysiology, prevention, and management and identify areas of further research.

## Incidence of postoperative delirium

Depending on the type of surgery, the incidence of POD is estimated to range from 5% to 65% in older surgical patients, with the highest incidence in cardiac and hip fracture repair surgery^4–6^. In one study, 19% of adults over age 60 experienced POD, suggesting close to 3 million elderly Americans are affected each year^1^. Delirium was also found to be an independent risk factor for long-term cognitive decline with subsequent progression to dementia, suggesting that POD might be a modifiable risk factor for dementia^7–9^. POD is preventable in up to 40% of patients and is a critical quality improvement target for preventative strategies in the perioperative setting^10^.

## Predictors of Delirium

It is essential to determine who is at risk for POD because only then can a plan be formulated to prevent it^11^. The risk of developing delirium is a combination of predisposing factors or vulnerabilities inherent to the patient and the precipitating factors or insults that occur in the perioperative period^1,12–14^. Preoperative organ dysfunction can lead to POD. Evidence also suggests a strong association between preoperative cognition, frailty, and mental disorders such as depression and anxiety. As suggested by Alam *et al.*, a combination of assessing cognition, frailty, and mental health disorders for older patients may confer a more robust approach to identifying patients at a higher risk of postoperative neurological complications^15^. With the emergence of enhanced recovery pathways, Jin *et al*. conducted a systematic review of the current clinical evidence for using delirium risk prediction scores, perioperative interventions for delirium risk reduction, and treatment options for established delirium. They established a link to the patient's age, type, and complexity of surgery and emergency surgeries. POD also led to prolonged hospital length of stay, increased 30-day mortality, significant functional decline, a two to three times higher risk of needing post-discharge care facilities, and increased healthcare costs^16^. The major predisposing risk factors and precipitating factors currently available in the literature are listed in Table 1^11,15–18^.

**Table 1.** Predisposing and Precipitating Factors.

Predisposing risk factors		Precipitating factors	
**Cognitive and behavior** ** disorders**	Prior history of delirium	**Surgery**	Type (hip fracture repair, cardiac, major vascular)
	Pre-existing cognitive impairment		Duration
	Sleep deprivation		Emergency surgery
	Depression		Blood loss/blood transfusion
	Alcohol abuse	**Anesthesia**	Hypotension
**Disease or illness related**	Frailty		Medications
	Sensory impairment (vision and hearing)	**Postoperative**	Poorly treated pain
	Functional impairment		Polypharmacy and high-dose opioids
	Multiple comorbidities		Use of physical restraints
	History of stroke		Bladder catheters
	Hereditary vulnerabilities—genetic		Infection
**Other**	Age over 65		Metabolic abnormalities
			ICU admission
			Prolonged mechanical ventilation
			Postoperative complications (e.g., infection, vascular events)

## Pathophysiology

The pathophysiology of POD is poorly understood. Research is ongoing to delineate the relevant pathways to identify targets for therapy. A neurotransmitter imbalance, namely an increase in acetylcholine and a decrease in dopamine, has been associated with POD. Neuroinflammation is also said to play a part as proteins have been identified that serve as risk markers and disease markers present at the time of delirium^19^. An increase in pro-inflammatory cytokines such as TNFα and IL-6 maintains a state of chronic inflammation manifesting as POD and postoperative cognitive dysfunction. However, there is still the need for robust well-powered preclinical and clinical studies to delineate the precise pathophysiology of POD^20^. Some studies suggest a blood-brain barrier (BBB) breakdown due to increased C-reactive protein and TNFα in POD patients^20–22^. Surgery, anesthesia, and an increase in IL-6 and mast cells cause reduced levels of tight junction proteins, thus disrupting the BBB. Mast cells also indirectly disrupt the BBB by breaking down the basal lamina^20^.

Current Alzheimer's disease research has led to the identification of novel biomarkers which may be beneficial in identifying high-risk patients. The functional brain connectivity changes in postoperative cognitive decline and Alzheimer's disease are similar. Damage to three major brain functional systems (i.e., medial temporal lobe/hippocampal memory system, diencephalon, and basal forebrain) can lead to amnesic syndrome^23^. The recently identified blood biomarker, plasma p-tau, provides insights into the biological basis of Alzheimer's disease, enabling neuropathological diagnosis several years earlier, estimating the future population burden, and developing therapeutic trials^24^. Liang *et al.* demonstrated an association between the preoperative plasma concentrations of tau, p-tau 217, and p-tau 181 and the incidence and severity of POD after adjusting for age, education, and preoperative Mini-Mental State Examination (MMSE). The group showed that in cardiac surgery, the levels peaked during cardiopulmonary bypass and remained elevated on postoperative days 1 and 2. The levels were also elevated in hip replacement surgery but not as dramatically as in cardiac surgery^25^. There may therefore be utility in exploring these biomarkers for POD. These biomarkers also have the potential to act as first-line diagnostic and prognostic tools that can offer the possibility of population screening for surgical patients. 

## Predicting

Preoperative cognition decline in older patients predicts POD and mortality^26,27^. Delirium risk is also greater for individuals with reduced MMSE scores on the delayed recall and working memory domains^28^. Learning and executive function are the key domains vulnerable to perioperative events. The Perioperative Brain Health Initiative recommends preoperative cognitive and delirium screening in at-risk patients using simple, short tests, including the Mini-Cog and 4 A's Test^29,30^. The Mini-Cog, MMSE, and Montreal Cognitive Assessment are widely used and have been shown to have good sensitivity and specificity. They are therefore recommended for preoperative assessment of cognition^13^. 

### Frailty syndrome

Frailty syndrome is an important geriatric syndrome characterized by age-associated declines in physiologic reserve and function in multiple organs, leading to increased vulnerability to stressors^31,32^. Not only is frailty associated with POD^33^, it is often associated with multimorbidity and a range of geriatric syndromes, including functional dependency, cognitive impairment, and malnutrition which further increase the risk and complexity of care. Clinical frailty scales are available as a screening tool in the preoperative period. In a study of the accuracy and feasibility of frailty instruments, the Clinical Frailty Scale was shown to have the largest pooled effect size for predicting mortality and non-home discharge after surgery and appeared to be the most practical instrument widely studied^34^.

## Prevention

Older patients presenting for surgery may come in with undiagnosed neurocognitive dysfunction, including Alzheimer's, Parkinson's, small vessel vascular dementia, and other neurodegenerative diseases^35^. Therefore, a multi-component intervention is effective in prevention.

### Multi-component Interventions

Prevention is the key to forestalling POD. Identifying patients with predisposing risk factors during the preoperative evaluation should include screening for potentially inappropriate medications linked with POD, such as benzodiazepines, sleeping aids, histamine type-2 receptor antagonists, first-generation antihistamines, skeletal muscle relaxants, gabapentinoids, and opioids. 

Screening for preoperative cognitive dysfunction which can be easily missed is essential^36^. Cognitive screening tools like digital testing are appropriate for preoperative evaluation clinics and fast-paced settings with limited staffing. The digital clock drawing test is feasible and highly acceptable to older adults in a preoperative setting^37^. For comparison, the Confusion Assessment Method for the ICU (CAM-ICU) delirium screener can be used as a baseline metric and repeated in the post-anesthesia care unit (PACU).

It is equally important to screen for malnutrition as a relationship between frailty syndrome and POD has been established. The geriatric nutrition risk index uses the patient's current weight, ideal body weight, and albumin to determine those at risk for malnutrition. It can predict POD and hospital length of stay in elderly patients undergoing noncardiac surgery^38^. The perioperative nutrition screen has also been used to implement preoperative nutrition interventions to optimize patients for surgery^39^. Frailty screening alone is inadequate and requires a multimodal approach that involves evaluation of the functional status, nutrition, and psychological state to evaluate if the patient can withstand the psychological stress of the perioperative period.

Anxiety and depression also predispose patients to POD. Therefore, measures will need to be in place to prepare patients for surgery psychologically. It is also vital to recognize the impact of social determinants of health on POD^40^. Alcohol withdrawal is a precipitating factor for delirium; reducing alcohol intake from four to two ounces may reduce the risk of perioperative complications by 50%. Once a high-risk patient has been identified that patient should be flagged to initiate a delirium prevention protocol.

## Prehabilitation

Enhancing an individual's functional capacity before a scheduled surgery, aimed at improving the patient's tolerance to the upcoming physiologic stress, is a significant area of research in preventing POD^41,42^. For instance, subjectively assessed functional capacity alone has uncertain accuracy and should not be used for preoperative risk evaluation. Clinicians should instead consider a measure such as the Duke Activity Status Index questionnaire score for cardiac risk assessment for major noncardiac surgeries^43^. Whether preoperative cognitive optimization is practical in older patients has undoubtedly been a concern; however, evidence suggests success is possible. Elderly patients who were at least minimally compliant in a tablet-based preoperative cognitive exercise program exhibited a lowered POD risk. Even though the amount of actual cognitive exercise time completed by patients varied widely, a significant difference was nevertheless demonstrated^44^.

One of the best prevention strategies currently is the Hospitalized Elder Life Program which has consistently prevented delirium in hospitalized older persons^45^. The structure involves a multidisciplinary team, daily visits, orientation, and ensuring the patient receives adequate sleep, nutrition, mobilization, and hydration. The ongoing Perioperative Cognitive Enhancement (PROTECT) Trial compares the multidisciplinary multi-component resource-heavy perioperative pathway to the current standard of care to reduce the incidence of delirium in elective surgical patients aged 65 years or older^46^. 

## Intraoperative

### Age-adjusted MAC and EEG-based anesthetic titration

Researchers using the Duke Anesthesia Resistance Scale (DARS) score, which combines age-adjusted minimum alveolar concentration (aaMAC) with bispectral index (BIS) score to determine POD risk, concluded that a score of less than 28.755 was associated with four-fold higher odds of POD. DARS is the average BIS divided by the quantity (2.5 minus the average aaMAC inhaled anesthetic fraction)^47^. Since a low DARS is independently associated with increased POD risk in older surgical patients, it is recommended that the aaMAC be used to adjust the end-tidal volatile anesthetic concentration.

### Lung-Protective Strategies

In a randomized, double-blind controlled trial of 64 elderly patients undergoing spinal surgery in the prone position, lung-protective strategies (low tidal volume, positive end-expiratory pressure of 5 cmH_2_O, and lung recruitment) significantly reduced the incidence of POD. The proposed mechanism for this positive result is reduced inflammation and improved cerebral oxygenation. The lung-protective strategy group also had lower levels of glial fibrillary acidic protein (GFAP) and IL-6, which have been correlated with delirium^48^. The study restricted participants to non-obese patients who had healthy lungs. Therefore, large-scale studies with less restrictive inclusion criteria are needed to apply the results broadly.

### Pharmacologic Agents

The consensus has been to avoid anticholinergics, benzodiazepines, and meperidine in high-risk patients. Dexmedetomidine is the most promising pharmacologic agent in reducing the incidence of POD in the elderly, critically ill, and ventilated patients^22,49,50^. Two new prospective studies revealed that dexmedetomidine infusions were associated with a decreased incidence of POD in thoracoscopic lobectomy and coronary artery bypass procedures^21,51^. It is nevertheless premature to suggest that dexmedetomidine may reduce POD. Other drugs that have been investigated include midazolam, glucocorticoids, and ramelteon. The use of midazolam in older patients is controversial because it is uncertain whether it has the potential for harm. A recent analysis of multiple prospective trials concluded that premedication with midazolam was not associated with early POD in older patients undergoing major noncardiac surgery^52^. Consequently, the risk of delirium associated with using a single-dose, short-acting agent such as midazolam is unclear. A recent Cochrane Review suggests that data are insufficient to determine whether benzodiazepines effectively treat delirium in non‐ICU settings^53^.

Although high-dose dexamethasone did not demonstrate any improvement in delirium occurrence in cardiac surgery in a small study^54^, a recent double-blind, randomized controlled trial revealed that patients receiving high-dose glucocorticoid had a lower occurrence of POD in the PACU 90 minutes after arrival and during the first four postoperative days^55^. Glucocorticoid use is promising, but more robust studies are needed to determine the dose and duration and the patient population most likely to benefit from it. Ramelteon, a melatonin receptor agonist, has been studied extensively; however, most studies have not demonstrated a benefit^56^.

Intraoperative anesthetic management should be individualized for older patients. It is critical to avoid intraoperative hypotension and wide swings in blood pressure. Intraoperative hypotension may put patients at risk of cerebral hypoperfusion with decreasing cerebral blood flow and is a modifiable risk factor that can be targeted to improve neurological outcomes^57^. While some studies have documented intraoperative and postoperative hypotension to be associated with delirium in critically ill patients^58^, others failed to confirm an association^59^. Normothermia should be maintained, as well as ensuring adequate analgesia and depth of anesthetic^60^. There is a lack of evidence that regional anesthetic techniques offer a protective effect against delirium^61,62^.

## Postoperative

Reorientation strategies to help patients familiarize themselves with their environment and the immediate care team should be the first line of care. Staff should introduce themselves to the patient and ensure minimal staff changes and transfers of care. Patients with hearing impairment should have access to their hearing aids, and those with vision impairment should be given their glasses. It is vital to ensure that the patient does not have pain, urinary retention, or constipation. Patients should have access to natural light and be well-hydrated^16^. While non-pharmacological interventions have been shown to decrease POD by 40%, in the immediate postoperative period, pharmacological agents are needed to calm the patient. Dexmedetomidine reduces astrocyte and microglial recruitment and inflammatory mediator expression in animal models. Evidence supports using dexmedetomidine, an α2 agonist, to reduce POD. However, to be effective, this involves continuous infusion throughout surgery and a few hours postoperatively^63^. Antipsychotics such as haloperidol have been used in the immediate postoperative period. Because of the incidence of complications, antipsychotics are not preferred. Restoring a normal sleep cycle by administering melatonin or ramelteon can normalize the circadian rhythm^16^.

## Conclusion

POD is an important clinical entity to recognize and diagnose because it can lead to significantly poorer outcomes. Brain and physical health are interconnected. The prevention of POD in the elderly requires a holistic multidisciplinary approach leveraging the entire perioperative continuum. Preventative strategies have been documented to prevent delirium in 40% of cases. Strategies include the detection of high-risk patients with a validated tool, the use of preventive non-pharmacological concepts, and preoperative evaluation to identify patients who may present with previously undiagnosed conditions that may impact delirium. Recognizing modifiable conditions and focusing on the most vulnerable patients is essential. Cognitive screening is necessary, and therefore the perioperative assessment should utilize simple tools to help identify patients who are at risk. These patients should be flagged, and steps should be taken to prevent POD. As POD is becoming more critical and evidence continues to emerge, it is hoped that hospitals will employ more resources to execute these efforts, which will require a multidisciplinary team and a robust preoperative service^64^. As more studies are conducted to understand the pathophysiology, plasma biomarkers have been found to be promising and may offer a more practical risk stratification method.

## References

[1] Watt J Tricco AC Talbot-Hamon C : Identifying Older Adults at Risk of Delirium Following Elective Surgery: A Systematic Review and Meta-Analysis. *J Gen Intern Med.* 2018; 33(4): 500–509. 10.1007/s11606-017-4204-x 29374358 PMC5880753

[2] Anand A MacLullich AMJ : Delirium in older adults. *Medicine.* 2021; 49(1): 26–31. 10.1016/j.mpmed.2020.10.002

[3] Evered L Silbert B Knopman DS : Recommendations for the nomenclature of cognitive change associated with anaesthesia and surgery-2018. *Anesthesiology.* 2018; 129(5): 872–879. 10.1097/ALN.0000000000002334 30325806

[4] Gleason LJ Schmitt EM Kosar CM : Effect of delirium and other major complications on outcomes after elective surgery in older adults. *JAMA Surg.* 2015; 150(12): 1134–1140. 10.1001/jamasurg.2015.2606 26352694 PMC4684425

[5] Boone MD Sites B von Recklinghausen FM : Economic burden of postoperative neurocognitive disorders among US Medicare patients. *JAMA Netw Open.* 2020; 3(7): e208931. 10.1001/jamanetworkopen.2020.8931 32735336 PMC7395237

[6] Mahanna-Gabrielli E Schenning KJ Eriksson LI : State of the clinical science of perioperative brain health: report from the American Society of Anesthesiologists Brain Health Initiative Summit 2018. *Br J Anaesth.* 2019; 123(4): 464–478. 10.1016/j.bja.2019.07.004 31439308 PMC6871269

[7] Inouye SK Marcantonio ER Kosar CM : The short-term and long-term relationship between delirium and cognitive trajectory in older surgical patients. *Alzheimers Dement.* 2016; 12(7): 766–775. 10.1016/j.jalz.2016.03.005 27103261 PMC4947419

[8] Goldberg TE Chen C Wang Y : Association of delirium with long-term cognitive decline: a meta-analysis. *JAMA Neurol.* 2020; 77(11): 1373–1381. 10.1001/jamaneurol.2020.2273 32658246 PMC7358977

[9] Fong TG Inouye SK : The inter-relationship between delirium and dementia: the importance of delirium prevention. *Nat Rev Neurol.* 2022; 18(10): 579–596. 10.1038/s41582-022-00698-7 36028563 PMC9415264

[10] Inouye SK Robinson T Blaum C : Postoperative delirium in older adults: best practice statement from the American Geriatrics Society. *J Am Coll Surg.* 2015; 220(2): 136–148. 10.1016/j.jamcollsurg.2014.10.019 25535170

[11] Lock S Chura L Barracca N : Preserving Your Brain Health During Illness or Surgery: GCBH Recommendations to Prevent and Treat Delirium. 2020. Reference Source

[12] Berger M Schenning KJ Brown CH 4th : Best practices for postoperative brain health: recommendations from the fifth International Perioperative Neurotoxicity Working Group. *Anesth Analg.* 2018; 127(6): 1406–1413. 10.1213/ANE.0000000000003841 30303868 PMC6309612

[13] Gracie TJ Caufield-Noll C Wang NY : The association of preoperative frailty and postoperative delirium: a meta-analysis. *Anesth Analg.* 2021; 133(2): 314–323. 10.1213/ANE.0000000000005609 34257192 PMC8289124

[14] Susano MJ Grasfield RH Friese M : Brief preoperative screening for frailty and cognitive impairment predicts delirium after spine surgery. *Anesthesiology.* 2020; 133(6): 1184–1191. 10.1097/ALN.0000000000003523 32898243 PMC7657972

[15] Alam A Ma D : Is it time to Assess Neurological Status Before Surgery to Improve Postoperative Outcomes? *Ann Surg.* 2022; 275(4): 644–645. 10.1097/SLA.0000000000005287 35129500

[16] Jin Z Hu J Ma D : Postoperative delirium: perioperative assessment, risk reduction, and management. *Br J Anaesth.* 2020; 125(4): 492–504. 10.1016/j.bja.2020.06.063 32798069

[17] Surgeons AC Surgeons AC : Optimal Resources for Geriatric Surgery: 2019 Standards. American College of Surgeons, 2019. Reference Source

[18] O’Gara BP Gao L Marcantonio ER : Sleep, Pain, and Cognition: Modifiable Targets for Optimal Perioperative Brain Health. *Anesthesiology.* 2021; 135(6): 1132–1152. 10.1097/ALN.0000000000004046 34731233 PMC8578455

[19] Vasunilashorn SM Ngo LH Chan NY : Development of a dynamic multi-protein signature of postoperative delirium. *J Gerontol A Biol Sci Med Sci.* 2019; 74(2): 261–268. 10.1093/gerona/gly036 29529166 PMC6333936

[20] Alam A Hana Z Jin Z : Surgery, neuroinflammation and cognitive impairment. *EBioMedicine.* 2018; 37: 547–556. 10.1016/j.ebiom.2018.10.021 30348620 PMC6284418

[21] Zhao J Wang WB Ding H : Prevention of Dexmedetomidine on Postoperative Delirium and Early Postoperative Cognitive Dysfunction in Elderly Patients Undergoing Thoracoscopic Lobectomy. *Evid Based Complement Alternat Med.* 2022; 2022: 5263021. 10.1155/2022/5263021 36276865 PMC9586721

[22] Vlisides P Avidan M : Recent Advances in Preventing and Managing Postoperative Delirium [version 1; peer review: 2 approved]. *F1000Res.* 2019; 8: F1000 Faculty Rev-607. 10.12688/f1000research.16780.1 31105934 PMC6498743

[23] Bauer RM Asken B : The three amnesias. In: *Textbook of clinical neuropsychology. * Taylor & Francis, 2017; 678–700. 10.4324/9781315271743-28

[24] Karikari TK Ashton NJ Brinkmalm G : Blood phospho-tau in Alzheimer disease: analysis, interpretation, and clinical utility. *Nat Rev Neurol.* 2022; 18(7): 400–418. 10.1038/s41582-022-00665-2 35585226

[25] Liang F Baldyga K Quan Q : Preoperative plasma tau-PT217 and tau-PT181 are associated with postoperative delirium. *Ann Surg.* 2022; 277(6): e1232–e1238. 10.1097/SLA.0000000000005487 35794069 PMC9875943

[26] Oresanya LB Lyons WL Finlayson E : Preoperative assessment of the older patient: a narrative review. *JAMA.* 2014; 311(20): 2110–2120. 10.1001/jama.2014.4573 24867014

[27] Arias F Bursian AC Sappenfield JW : Delirium history and preoperative mild neurocognitive disorder: an opportunity for multidisciplinary patient-centered care. *Am J Case Rep.* 2018; 19: 1324–1328. 10.12659/AJCR.911437 30397190 PMC6232917

[28] Price CC Garvan C Hizel LP : Delayed recall and working memory MMSE domains predict delirium following cardiac surgery. *J Alzheimers Dis.* 2017; 59(3): 1027–1035. 10.3233/JAD-170380 28697572 PMC5544543

[29] Limpawattana P Manjavong M : The Mini-Cog, Clock Drawing Test, and Three-Item Recall Test: Rapid Cognitive Screening Tools with Comparable Performance in Detecting Mild NCD in Older Patients. *Geriatrics (Basel).* 2021; 6(3): 91. 10.3390/geriatrics6030091 34562992 PMC8482262

[30] MacLullich AM Shenkin SD Goodacre S : The 4 'A's test for detecting delirium in acute medical patients: a diagnostic accuracy study. *Health Technol Assess.* 2019; 23(40): 1–194. 10.3310/hta23400 31397263 PMC6709509

[31] De Biasio JC Mittel AM Mueller AL : Frailty in critical care medicine: a review. *Anesth Analg.* 2020; 130(6): 1462–1473. 10.1213/ANE.0000000000004665 32384336 PMC7426653

[32] Miyamura K Fhon JRS Bueno AA : Frailty syndrome and cognitive impairment in older adults: systematic review of the literature. *Rev Lat Am Enfermagem.* 2019; 27: e3202. 10.1590/1518-8345.3189.3202 31664410 PMC6818658

[33] Mahanna-Gabrielli E Zhang K Sieber FE : Frailty Is Associated With Postoperative Delirium But Not With Postoperative Cognitive Decline in Older Noncardiac Surgery Patients. *Anesth Analg.* 2020; 130(6): 1516–1523. 10.1213/ANE.0000000000004773 32384341 PMC7875454

[34] Aucoin SD Hao M Sohi R : Accuracy and feasibility of clinically applied frailty instruments before surgery: a systematic review and meta-analysis. *Anesthesiology.* 2020; 133(1): 78–95. 10.1097/ALN.0000000000003257 32243326

[35] Weuve J Hebert LE Scherr PA : Prevalence of Alzheimer disease in US states. *Epidemiology.* 2015; 26(1): e4–e6. 10.1097/EDE.0000000000000199 25437325

[36] Peden CJ Miller TR Deiner SG : Improving perioperative brain health: an expert consensus review of key actions for the perioperative care team. *Br J Anaesth.* 2021; 126(2): 423–432. 10.1016/j.bja.2020.10.037 33413977

[37] Buckley RA Atkins KJ Fortunato E : A novel digital clock drawing test as a screening tool for perioperative neurocognitive disorders: A feasibility study. *Acta Anaesthesiol Scand.* 2021; 65(4): 473–480. 10.1111/aas.13756 33296501

[38] Zhao Y Xia X Xie D : Geriatric Nutritional Risk Index can predict postoperative delirium and hospital length of stay in elderly patients undergoing non-cardiac surgery. *Geriatr Gerontol Int.* 2020; 20(8): 759–764. 10.1111/ggi.13963 32570290 PMC7496996

[39] Williams DG Aronson S Murray S : Validation of the perioperative nutrition screen for prediction of postoperative outcomes. *JPEN J Parenter Enteral Nutr.* 2022; 46(6): 1307–1315. 10.1002/jpen.2310 34850403

[40] Magnan S : Social determinants of health 101 for health care: five plus five. *NAM perspectives.* 2017. 10.31478/201710c PMC8406598 34532697

[41] Norris CM Close JCT : Prehabilitation for the frailty syndrome: improving outcomes for our most vulnerable patients. *Anesth Analg.* 2020; 130(6): 1524–1533. 10.1213/ANE.0000000000004785 32384342

[42] Kow AW : Prehabilitation and its role in geriatric surgery. *Ann Acad Med Singap.* 2019; 48(11): 386–392. 10.47102/annals-acadmedsg.V48N11p386 31960020

[43] Wijeysundera DN Pearse RM Shulman MA : Assessment of functional capacity before major non-cardiac surgery: an international, prospective cohort study. *Lancet.* 2018; 391(10140): 2631–2640. 10.1016/S0140-6736(18)31131-0 30070222

[44] Humeidan ML Reyes JPC Mavarez-Martinez A : Effect of cognitive prehabilitation on the incidence of postoperative delirium among older adults undergoing major noncardiac surgery: the neurobics randomized clinical trial. *JAMA Surg.* 2021; 156(2): 148–156. 10.1001/jamasurg.2020.4371 33175114 PMC7658803

[45] Hshieh TT Yang T Gartaganis SL : Hospital elder life program: systematic review and meta-analysis of effectiveness. *Am J Geriatr Psychiatry.* 2018; 26(10): 1015–1033. 10.1016/j.jagp.2018.06.007 30076080 PMC6362826

[46] Atkins KJ Scott DA Silbert B : Preventing Delirium and Promoting Long-Term Brain Health: A Clinical Trial Design for the Perioperative Cognitive Enhancement (PROTECT) Trial. *J Alzheimers Dis.* 2021; 83(4): 1637–1649. 10.3233/JAD-210438 34420958

[47] Cooter Wright M Bunning T Eleswarpu SS : A Processed Electroencephalogram–Based Brain Anesthetic Resistance Index Is Associated With Postoperative Delirium in Older Adults: A Dual Center Study. *Anesth Analg.* 2022; 134(1): 149–158. 10.1213/ANE.0000000000005660 34252066 PMC8678136

[48] Wang J Zhu L Li Y : The potential role of lung-protective ventilation in preventing postoperative delirium in elderly patients undergoing prone spinal surgery: a preliminary study. *Med Sci Monit.* 2020; 26: e926526. 10.12659/MSM.926526 33011734 PMC7542993

[49] Duan X Coburn M Rossaint R : Efficacy of perioperative dexmedetomidine on postoperative delirium: systematic review and meta-analysis with trial sequential analysis of randomised controlled trials. *Br J Anaesth.* 2018; 121(2): 384–397. 10.1016/j.bja.2018.04.046 30032877

[50] Su X Meng ZT Wu XH : Dexmedetomidine for prevention of delirium in elderly patients after non-cardiac surgery: a randomised, double-blind, placebo-controlled trial. *Lancet.* 2016; 388(10054): 1893–1902. 10.1016/S0140-6736(16)30580-3 27542303

[51] Singh A Garg V Mehta Y : Perioperative dexmedetomidine reduces delirium after coronary artery bypass graft surgery: A prospective, single-blind, observational study. *Ann Card Anaesth.* 2022; 25(4): 490–497. 10.4103/aca.aca_45_21 36254916 PMC9732957

[52] Wang ML Min J Sands LP : Midazolam premedication immediately before surgery is not associated with early postoperative delirium. *Anesth Analg.* 2021; 133(3): 765–771. 10.1213/ANE.0000000000005482 33721875 PMC8373629

[53] Li Y Ma J Jin Y : Benzodiazepines for treatment of patients with delirium excluding those who are cared for in an intensive care unit. *Cochrane Database Syst Rev.* 2020; 2(2): CD012670. 10.1002/14651858.CD012670.pub2 32108325 PMC7047222

[54] Sauër AMC Slooter AJC Veldhuijzen DS : Intraoperative dexamethasone and delirium after cardiac surgery: a randomized clinical trial. *Anesth Analg.* 2014; 119(5): 1046–1052. 10.1213/ANE.0000000000000248 24810262

[55] Awada HN Steinthorsdottir KJ Schultz NA : High‐dose preoperative glucocorticoid for prevention of emergence and postoperative delirium in liver resection: A double‐blinded randomized clinical trial substudy. *Acta Anaesthesiol Scand.* 2022; 66(6): 696–703. 10.1111/aas.14057 35325467 PMC9320957

[56] Oh ES Leoutsakos JM Rosenberg PB : Effects of ramelteon on the prevention of postoperative delirium in older patients undergoing orthopedic surgery: the RECOVER randomized controlled trial. *Am J Geriatr Psychiatry.* 2021; 29(1): 90–100. 10.1016/j.jagp.2020.05.006 32532654 PMC8809889

[57] Yu Q Qi J Wang Y : Intraoperative hypotension and neurological outcomes. *Curr Opin Anaesthesiol.* 2020; 33(5): 646–650. 10.1097/ACO.0000000000000904 32769747

[58] Maheshwari K Ahuja S Khanna AK : Association between perioperative hypotension and delirium in postoperative critically ill patients: a retrospective cohort analysis. *Anesth Analg.* 2020; 130(3): 636–643. 10.1213/ANE.0000000000004517 31725024

[59] Langer T Santini A Zadek F : Intraoperative hypotension is not associated with postoperative cognitive dysfunction in elderly patients undergoing general anesthesia for surgery: results of a randomized controlled pilot trial. *J Clin Anesth.* 2019; 52: 111–118. 10.1016/j.jclinane.2018.09.021 30243062

[60] Duning T Ilting-Reuke K Beckhuis M : Postoperative delirium – treatment and prevention. *Curr Opin Anaesthesiol.* 2021; 34(1): 27–32. 10.1097/ACO.0000000000000939 33315641

[61] Ilango S Pulle RC Bell J : General versus spinal anaesthesia and postoperative delirium in an orthogeriatric population. *Australas J Ageing.* 2016; 35(1): 42–47. 10.1111/ajag.12212 26364948

[62] Neuman MD Feng R Carson JL : Spinal anesthesia or general anesthesia for hip surgery in older adults. *N Engl J Med.* 2021; 385(22): 2025–2035. 10.1056/NEJMoa2113514 34623788

[63] Zeng H Li Z He J : Dexmedetomidine for the prevention of postoperative delirium in elderly patients undergoing noncardiac surgery: A meta-analysis of randomized controlled trials. *PLoS One.* 2019; 14(8): e0218088. 10.1371/journal.pone.0218088 31419229 PMC6697366

[64] Ishizawa Y : Does Preoperative Cognitive Optimization Improve Postoperative Outcomes in the Elderly? *J Clin Med.* 2022; 11(2): 445. 10.3390/jcm11020445 35054139 PMC8778093

